# The effects of stimulation waveform and carrier frequency on tolerance and motor thresholds elicited by transcutaneous spinal cord stimulation in stroke

**DOI:** 10.1016/j.cnp.2025.04.001

**Published:** 2025-04-22

**Authors:** Chen Yang, Nicole C. Veit, Kelly A. McKenzie, Shreya Aalla, Ameen Kishta, Kyle Embry, Elliot J. Roth, Richard L. Lieber, Arun Jayaraman

**Affiliations:** a Shirley Ryan AbilityLab, Chicago, IL 60611, USA; b Feinberg School of Medicine, Northwestern University, Chicago, IL 60611, USA; c Biomedical Engineering Department, McCormick School of Engineering, Northwestern University, Evanston, IL 60208, USA; d Hines VA Medical Center, Maywood, IL 60141, USA

**Keywords:** Stimulation parameters, Russian current, Modulation frequency, Motor rehabilitation, Neuromodulation, EMG

## Abstract

**Objective:**

With growing interest in translating transcutaneous spinal cord stimulation (tSCS) into rehabilitation for different neurologic injuries, understanding the effects of various combinations of stimulation parameters becomes essential.

**Methods:**

Twenty-one participants post-stroke completed an assessment to determine their resting motor threshold (RMT) (minimum current required to elicit a muscle response) and tolerance levels (uncomfortable current intensity) to 12 stimulation configurations: two square waveforms, biphasic and monophasic, paired with six carrier frequencies (unmodulated: 0, and modulated: 1, 3, 5, 7, and 10 kHz).

**Results:**

The results demonstrated that increasing carrier frequency increased participants’ tolerance level and RMTs. Carrier frequency nor waveform type significantly altered discomfort when tolerance was normalized to the motor threshold, with 57 ± 23 % tolerated across all configurations. However, higher carrier frequencies, particularly biphasic waveforms at frequencies > 5 kHz, required more charge to reach a muscle activation and activated fewer muscles compared to unmodulated waveforms. No significant differences in discomfort relative to RMT were found between monophasic and biphasic waveforms.

**Conclusions:**

Higher carrier frequency allows stimulation to be more comfortable at a given intensity, but it also requires more current to reach RMTs.

**Significance:**

This study provides an essential feasibility assessment of tSCS configurations in a neurological population.

## Highlights

• First study examining transcutaneous spinal cord stimulation (tSCS) carrier frequencies and waveforms in stroke survivors.
• Tolerance to tSCS is consistent across carrier frequencies and waveforms when normalized to motor threshold.
• Matching total charge delivered across tSCS parameters does not ensure equivalent muscle output.

## 1 Introduction

Transcutaneous spinal cord stimulation (tSCS) has gained attention in neurorehabilitation, particularly for its non-invasive nature, affordability, and ease of use, making it well-accepted by patients. While tSCS has been primarily studied in individuals with spinal cord injury (SCI) (Estes et al., 2021, Gad et al., 2017, McHugh et al., 2020, Shapkova et al., 2020), it has recently been explored in stroke gait rehabilitation, where it has shown promise in improving walking speed, gait quality, and coordination when combined with gait training (Bogacheva et al., 2023, Moon et al., 2024, Moshonkina et al., 2022, Skvortsov et al., 2023).

Applying tSCS in individuals post-stroke presents unique challenges, particularly when determining appropriate stimulation dosage and ensuring participant comfort. In our previous studies, we used individual subject’s resting motor threshold (RMT), defined as the minimum current required to elicit a muscle response measured by EMG, as a physiologic reference to set stimulation intensity as a percentage of RMT during a gait intervention (McKenzie et al., 2024, Moon et al., 2024). This approach allows for objective dosage quantification, ensures consistency across participants, and helps avoid involuntary muscle contractions during walking by ensuring subthreshold stimulation. Additionally, tSCS may cause discomfort at the stimulation site, particularly as high currents are required to pass multiple layers of tissue to reach the spinal cord. This issue is especially relevant in individuals post-stroke, who often retain more sensation in their back than those with SCI, increasing the likelihood of discomfort and limiting clinical feasibility.

Both RMT and comfort can be influenced by parameters such as waveform and carrier frequency, which shape how electrical current is delivered to the spinal cord. Waveform refers to the shape of the electrical pulse, with common types including monophasic (unidirectional) and biphasic (alternating positive and negative phases). Carrier frequency involves embedding high-frequency pulses within a single stimulation pulse, a technique proposed to modify neural activation and improve comfort (Gad et al., 2018, Gerasimenko et al., 2015). A recent systematic review (Laskin et al., 2022) found that 15 of 24 tSCS studies used biphasic waveforms, while 9 used monophasic waveforms, but carrier frequencies were inconsistently reported. A lack of guidelines and understanding of waveforms and frequencies hinders the comparability, generalizability, and clinical application of tSCS, particularly as it is translated to other neurological populations such as stroke.

To address these challenges, we investigated the impact of waveform and carrier frequency on RMTs and tolerability with the goal of informing stimulation protocols that may be more feasible in the stroke population and offering insights into future study designs. This study aimed to systematically examine how waveform and carrier frequency affect RMT, tolerance levels, charge delivered, and muscle activation. We hypothesized that the variations in waveform and carrier frequency will result in changes in charge delivered, subsequently affecting RMT and tolerance level.

## 2 Methods

### 2.1 Participants

This study was conducted at Shirley Ryan AbilityLab in Chicago, Illinois. The trial protocol was approved by Northwestern University Institutional Review Board (STU00215009) and preregistered at ClinicalTrials.gov (NCT05167786). All participants provided written informed consent. The study design and conduct complied with all relevant regulations regarding the use of human study participants and was conducted in accordance with the criteria set by the Declaration of Helsinki.

Twenty-one participants (Table 1) were recruited from the Shirley Ryan AbilityLab via convenience sampling. Inclusion criteria were: age > 18 years, ≥6 months post-stroke, hemiplegia from a single stroke, Functional Ambulation Category ≥ 2, ability to provide informed consent, no current physical therapy, and physician approval. Exclusion criteria included: ataxia, multiple strokes, recent botulinum toxin injection (<4 months), severe spasticity (Modified Ashworth Scale ≥ 3), pregnancy, pacemaker, active pressure sores, unhealed fractures, peripheral neuropathy, painful musculoskeletal conditions, severe contractures, medical conditions limiting walking, active UTIs, significant psychiatric or substance abuse issues, metal spinal implants, and active or recent (<5 years) cancer.

**Table 1:** Participants’ demographics.

**ID**	**Sex**	**Age (yrs)**	**Chronicity (yrs)**	**Stroke Type**	**Height (cm)**	**Weight (kg)**	**SSV (m/s)**
1	F	59	5.9	Isch	162	82.7	0.77
2	M	56	9.7	Isch	152	118.2	0.96
3	M	70	9.0	Hemo	173	77.3	0.27
4	M	55	5.7	Isch	173	73.6	0.46
5	M	57	9.0	Isch	168	66.4	0.68
6	M	54	2.9	Isch	175	80.5	1.08
7	M	71	6.0	Hemo	175	61.4	0.09
8	M	63	7.8	Isch	190	123.2	0.79
9	M	52	6.2	Hemo	182	115.9	1.10
10	M	48	7.9	Hemo	172	76.4	0.67
11	M	49	1.1	Hemo	175	104.5	1.56
12	F	64	1.8	Hemo	160	84.1	0.37
13	M	41	2.2	Hemo	173	79.4	0.80
*14	F	49	6.3	Hemo	162	76.8	0.74
*15	F	63	8.3	Isch	160	72.7	0.92
*16	F	57	4.8	Hemo	170	70.5	1.00
*17	F	65	4.1	Isch	158	95.3	1.00
*18	F	69	5.6	Hemo	157	59.1	0.40
*19	F	54	1.0	Isch	170	88.6	1.18
*20	M	65	4.7	Isch	170	82.7	0.32
*21	F	70	2.5	Isch	165	110.0	0.83

* The last 8 participants listed did not achieve activation of at least 4 muscles in the unmodulated (0 kHz) waveform due to the low tolerance during the spinal motor evoked responses tests. Isch: Ischemic; Hemo: Hemorrhagic. SSV = self-selected velocity.

### 2.2 Study protocol

In this study, two waveforms, biphasic and monophasic, were paired with carrier frequencies: unmodulated 0 kHz, and modulated 1 ms pulses with 1 kHz, 3 kHz, 5 kHz, 7 kHz, and 10 kHz, resulting in a total of 12 stimulation waveform-frequency configurations. A constant current stimulator (DS8R, Digitimer Ltd., UK) was used and triggered by a customized control unit for the delivery of modulated pulses with carrier frequencies. Configurations are abbreviated the text using ‘B’ or ‘M’ for waveform followed by the carrier frequency: B0 for biphasic at 0 kHz, M10 for monophasic at 10 k Hz, etc. All configurations had a total pulse width of 1 ms, except B0 with a total pulse width was 2 ms and M1 with a total pulse of 0.5 ms, this way the negative phase duration was the same between monophasic and biphasic waveforms within each carrier frequency. Each participant completed a neurophysiological assessment to obtain the motor threshold referred as the “spinal motor evoked responses (sMER) test” and tolerance to continuous stimulation evaluation for each configuration. Each session consisted of 4 sMER tests and 4 tolerance tests, with the tolerance and sMER for a given configuration always tested on the same day. There was a minimum 2-day washout period between visits.

#### 2.2.1 Spinal motor evoked responses (sMERs) test

**Fig. 1:**
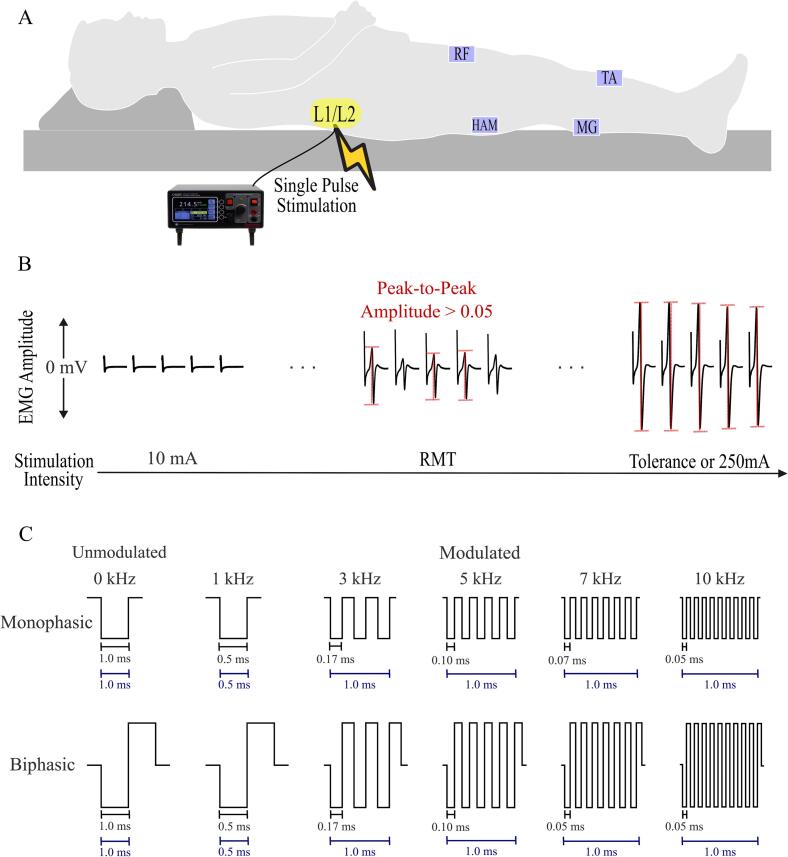
Neurophysiological assessment (referred to as the sMERs test) to record spinal motor evoked muscle responses (sMERs) for each waveform-frequency configuration. A) Schematic of the protocol setup to perform spinal motor evoked responses. Stimulation was delivered at L1/L2 vertebral levels as single pulses while participant laid supine and relaxed. EMG activity was recorded from four muscles bilaterally: RF, HAM, TA, MG. B) Example of evoked responses (sMERs) measured by EMG for a single muscle at increasing intensities. Five stimulation pulses where delivered for each current intensity. The RMT was defined as the current intensity needed to elicit a muscle response greater than 0.05 mV in 3 out of the 5 trials tested. The sMERs test was performed up to single-pulse tolerance or capped at 250 mA for all stimulation configurations. (C) The twelve stimulation waveform-frequency configurations tested in the study. The figure shows the duration of a single negative phase of each configuration (black line), calculated as 0.5*1ms/(Frequency in kHz). The total pulse width (defined as negative plus positive phases) was set to 1 ms for all configurations, except for biphasic unmodulated (BU), where the total pulse width was 2 ms, and monophasic 1 kHz with a total pulse width of 0.5 ms (blue line). This adjustment ensured a consistent negative phase duration between monophasic and biphasic configurations at the same carrier frequency. RMT: resting motor threshold, RF: rectus femoris, HAM: biceps femoris, TA: tibialis anterior, MG: medial gastrocnemius.

To investigate how different parameter configurations of tSCS affect muscle activation and motor thresholds, we performed a neurophysiological assessment that involved delivering stimulation at increasing intensities and recording the electrical activity of the muscles with EMG electrodes. This recorded muscle response, which is the compound muscle action potential, is referred to as a “spinal motor evoked response” (sMER). And therefore, the protocol to obtain these muscle responses (e.g. sMERs) at various current intensities is referred to as the sMERs test. We performed the sMERs test for each waveform-frequency configuration to determine the resting motor threshold (RMT) of leg muscles (Fig. 1). A single 3.2 cm diameter electrode (ValuTrode, Axelgaard Ltd., Fallbrook, CA, USA) was placed medially between the L1 and L2 spinous processes. Additionally, a pair of electrodes (UltraSim, Axelgaard Ltd., 7.5 × 13 cm, Fallbrook, CA, USA) was placed symmetrically over the iliac crests. The spine electrode acted as the cathode for monophasic waveforms, and it was the cathode in the first phase and anode in the second phase for biphasic waveforms. After electrode placement, participants were asked to lie in a relaxed, supine position and to avoid limb movements during testing. Stimulation was delivered as a single pulse at 10 mA intensity increments, increasing from 10 mA to 250 mA or until the participant reached maximum tolerance. The duration of each pulse for each configuration is shown in Fig. 1C. Participants’ tolerance in this test was recorded as the ***single-pulse tolerance.*** Each stimulation intensity was delivered five times in 5 s intervals (Gerasimenko et al., 2015, Minassian et al., 2007). Surface EMG activity was recorded with bipolar Ag-AgCl surface electrodes (GS26, Bio-Medical Instruments, Clinton Charter Township, MI, USA). Each electrode pair was placed longitudinally on the belly of the muscles of rectus femoris (RF), medial hamstring (HAM), tibialis anterior (TA), and medial gastrocnemius (MG) on both legs. Ground reference electrodes were placed bilaterally over the bony prominence of the patella. EMG signals were sampled at 4000 Hz using PowerLab 16/35 data acquisition system operated with LabChart software (v7.2, AD Instruments, Bella Vista, NSW, Australia). The sMER test was conducted for all 12 stimulation parameter configurations in random order, with a maximum of 4 configurations tested per day and participants blinded. Randomization was performed by a study member using a custom MATLAB code. Before each session, we documented tSCS and EMG electrode placement by photographs and recorded precise measurements using bony landmarks to ensure consistency across multiple sessions for each participant.

#### 2.2.2 Continuous stimulation tolerance testing

In addition to the sMER testing, participants underwent a tolerance level assessment to continuous stimulation for each configuration. Each participant was asked to walk on the treadmill at their comfortable speed while receiving continuous stimulation delivered at 30 Hz for a specific configuration. During walking, the stimulation intensity was gradually increased until participants reported it was too intense. From pilot testing, we observed that tolerance improved after a 2-minute habituation period. In the current study, once the initial tolerance threshold was reached, intensity was reduced by 10 %, and participants continued walking for two minutes before the intensity was gradually increased again. This recorded stimulation intensity was considered the ***continuous stimulation tolerance*** for the specific stimulation configuration. The tolerance test was conducted for all the configurations in random order, with a 5-minute rest period between testing, and a maximum of four configurations tested per day. The cathode/anode electrodes placement and pulse widths remained consistent with the sMER testing.

### 2.3 Data analysis

#### 2.3.1 Resting motor threshold (RMT), continuous stimulation tolerance, and percent of RMT tolerated

EMG responses to single-pulse stimulation obtained during sMER tests was initially bandpass-filtered (fourth-order Bessel filter, 30–2000 Hz) by the PowerLab 16/35 data acquisition system operated with LabChart software (v7.2, AD Instruments, Bella Vista, NSW, Australia). Afterwards, the EMG data was low pass filtered (5th order low-pass Butterworth, 450 Hz) using Matlab (Matworks Inc, USA). The resting motor threshold (RMT) for each muscle was defined as the minimal current intensity required to elicit a compound muscle action potential with a peak-to-peak amplitude of 0.05 mV in 3 out of 5 consecutive trials (Fig. 1B). For each stimulation configuration, the mean of the RMT values across the aforementioned 8 muscles were obtained to determine an ***average RMT*** value, as this could represent the reference current intensity used for a stimulation intervention. In addition, we included supplementary analysis of the ***lowest RMT*** by selecting the muscle with lowest RMT value out of the eight muscles and have a reference for a true subthreshold value, since an intensity delivered above this level may cause an involuntary muscle contraction in that muscle. For each configuration, we calculated the percent of RMT that could be tolerated for both average RMT and lowest RMT as follows:

$$R_{i}=\frac{\mathrm{Continous}\;{\mathrm{tolerance}}_{i}}{{\mathrm{RMT}}_{i}}\times 100\%$$

i represents the ith tested configuration. Therefore, the configuration with the highest percent of RMT tolerated may be considered as the most comfortable and effective if the goal would be to deliver stimulation closest to the RMT.

#### 2.3.2 Number of activated muscles

Furthermore, we calculated how many muscles reached the RMT level by the end of the sMER test for each stimulation configuration. Eight of the twenty-one participants were defined as “non-responders” since they did not have a muscle response (no RMT reached) for at least 4 out of 8 muscles in the 0 kHz configurations. The data for these participants were used only for the analysis of tolerance and total number of muscles activated and excluded from the analysis of RMT and percent of RMT tolerated.

#### 2.3.3 Charge delivered at the RMT intensity

We also present the RMT in units of electrical charge (uC) to further explore the differences between each configuration. The total charge delivered was calculated as follows:

For unmodulated 0 kHz configuration the following equation was used:

(1) $$Total\;charge\;\left(uC\right)\;=\;total\;pulse\;width\;(1\;ms)\;\times \;RMT\;intensity\;\left(mA\right)$$

For modulated 1 kHz to 10 kHz configurations, the following equation was used:

(2) $$Total\;charge\;\left(uC\right)\;=\;50\%\;duty\;cycle\;\times \;total\;pulse\;width\;(1ms)\;\times \;RMT\;intensity\;\left(mA\right)$$

For biphasic pulses, both equations, only account for the charge delivered at cathodic (negative) phase. At a constant current, equation (2) yields the same charge delivered all configuration 1 to 10 kHz since the total pulse width was 1 ms and all had a duty cycle of 50 % (positive and negative phases of the pulse were the same duration).

### 2.4 Statistical analysis

All outcomes (i.e., RMTs, single-pulse tolerances, continuous tolerances, percent of RMT tolerated, number of activated muscles, and total charge delivered at RMT) were first assessed for normality using Shapiro-Wilk Tests. Generalized estimating equations (GEEs; Link Function = Identity; Structure of Covariance Matrix = Exchangeable) were conducted to test the effects of waveform (monophasic vs. biphasic) and carrier frequency (0 kHz vs. 1 kHz vs. 3 kHz vs. 5 kHz vs.7 kHz vs. 10 kHz) on all the outcomes. Interaction effects were examined by post-hoc pairwise comparisons with Sequential Bonferroni adjustments and significance level set to α < 0.05. Only significant interaction pairwise comparisons referring to differences between waveforms within each carrier frequency, or differences between carrier frequencies within each waveform are presented in the results section, and complete post-hoc results for significant interaction effects are presented in the supplementary tables. GEE was selected because it obtains higher power with a small sample size compared to repeated measured analysis of variance, and it provided unbiased estimates with missing data (Ma et al., 2012, Naseri et al., 2016).

The statistical analysis for single-pulse tolerance, continuous stimulation tolerance, and number of muscles activated was performed for all the participants (n = 21). The statistical analysis for the rest of the outcomes was performed only for the responders (n = 13). We conducted a GEE analysis to explore differences between responders and non-responders in single-pulse and continuous stimulation tolerance, using group (responders vs. non-responders), carrier frequency, and waveform as independent variables. All results are presented as median and interquartile range (IQR).

## 3 Results

### 3.1 Resting motor threshold (RMT)

**Fig. 2:**
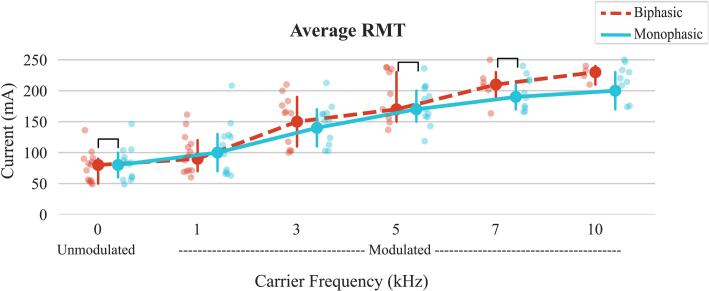
Average resting motor thresholds (RMTs) for each waveform-carrier frequency configuration. Brackets indicate a significant difference between monophasic and biphasic at a certain carrier frequency. The plot shows the median values, with error bars representing the interquartile range (IQR), covering the 25th to 75th percentiles of the data.

For each of the 12 configurations tested, the average RMT (across 8 muscles) increased as carrier frequency increased, demonstrating a significant carrier frequency effect (p < 0.001). Biphasic RMTs were significantly greater than monophasic RMTs across all carrier frequencies (p < 0.001). Additionally, a significant waveform-carrier frequency interaction effect was observed for average RMT (p < 0.001). There were significant differences between biphasic and monophasic RMTs with 0 kHz, 5 kHz, and 7 kHz carrier frequencies (B0: median 80.0 mA (IQR: 55.0–90.0) vs. M0: 83.8 mA (60.0–92.5), p = 0.012; B5: 195.0 mA (156.2–235.0) vs. M5: 178.1 mA (158.4–204.2), p < 0.001; B7: 210.2 mA (202.9–218.3) vs. M7: 192.4 mA (173.1–217.4), p = 0.001), with average RMTs higher in biphasic compared to monophasic at both 5 kHz and 7 kHz (Fig. 2). Average RMTs for each waveform-frequency configuration for all participants are shown in Supplementary Table S1, and all pairwise comparisons are shown in Supplementary Table S2.

### 3.2 Tolerance to stimulation

**Fig. 3:**
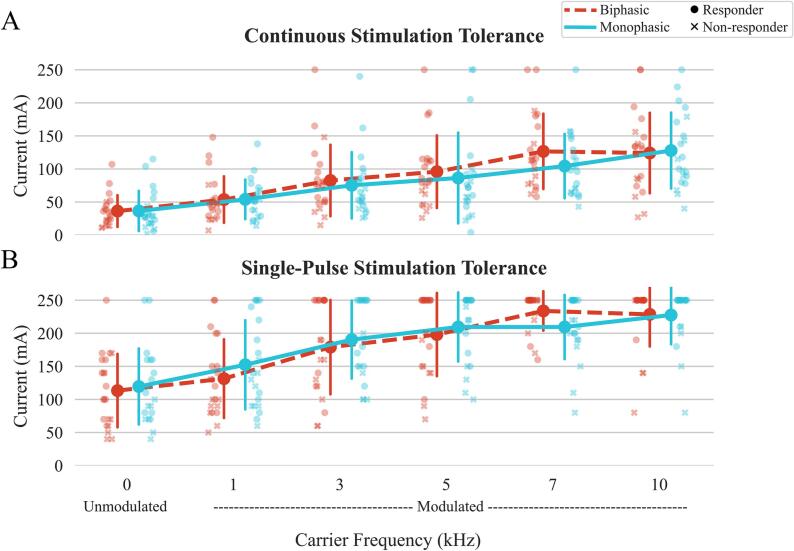
(A) Continuous stimulation tolerance obtained as participants walked on the treadmill at a comfortable speed while stimulation was delivered at 30 Hz and (B) single-pulse stimulation tolerance obtained from the sMERs assessment for each waveform-carrier frequency configuration. The plot shows the median values, with error bars representing the interquartile range (IQR), covering the 25th to 75th percentiles of the data. Eight of the twenty-one participants that were defined as “non-responders” since they did not have a muscle response (no RMT reached) for at least 4 out of 8 muscles in the 0 kHz configurations are marked with an “x”.

For continuous stimulation tolerance intensity (Fig. 3A), there was a significant carrier frequency main effect (p < 0.001) and a significant waveform main effect (p = 0.038). The tolerance intensity increased with carrier frequency regardless of waveform (0 kHz: median 28.5 mA (IQR: 18.0–44.8), 10 kHz: 123.0 mA (84.5–154.5)). Regardless of carrier frequency, the tolerance intensity of biphasic waveform was significantly higher than it was of monophasic waveform (biphasic: 75.5 mA (42.5–116.0) vs. monophasic: 66.5 mA (40.0–100.8)). However, the interaction effect was not significant (p = 0.151), suggesting that tolerances between specific waveform-frequency configurations did not differ. The maximum tolerance intensities per participant for each waveform-frequency combination is presented in Supplementary Table S3. In addition, participants’ tolerance increased after 2 min of habituation by 14.4 ± 2.7 (mean ± standard deviation) % across all biphasic configurations and 18.5 ± 5.0 % across all monophasic configurations.

For single-pulse tolerance (Fig. 3B), there was a significant carrier frequency effect (p < 0.001). Overall, participants tolerated absolute higher intensities with higher carrier frequency no matter which waveform in the sMER tests (0 kHz: 105 mA (70–160), 10 kHz: 250 mA (250–250)). No significant differences were found between waveforms (biphasic vs monophasic) within the same carrier frequency, or between carrier frequencies within the same waveform.

### 3.3 Percent of RMT tolerated

**Fig. 4:**
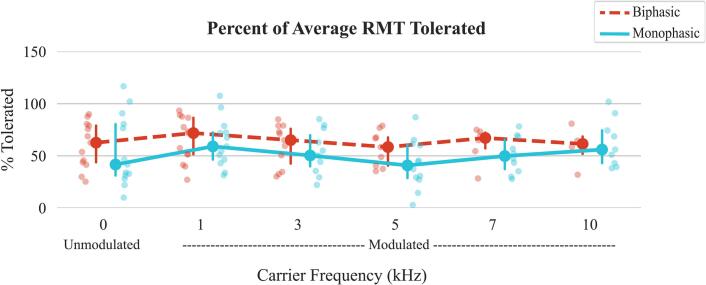
Percent of average RMT tolerated for each waveform-carrier frequency configuration. This percentage reflects the maximum stimulation intensity participants could tolerate, relative to their own RMT—a physiological measure representing the current needed to elicit a muscle response. The plot shows the median values, with error bars representing the interquartile range (IQR), covering the 25th to 75th percentiles of the data.

The percent of average RMT tolerated was calculated to normalize discomfort relative to the intensity required for muscle activation across waveform-frequency configurations. Significant interaction effects were observed for the percent of average RMT tolerated (Fig. 4, p = 0.005). However, significant pairwise comparisons occurred only between configurations with different waveforms and carrier frequencies (see Supplementary Table S4). No significant differences were found between waveforms at the same carrier frequency or between carrier frequencies within a single waveform.

On average, the percent of average RMT tolerated across all configurations of biphasic was 61 ± 20 % (median 65 % (IQR: 43–77)) and monophasic 54 ± 25 % (median 50 % (IQR: 35–72)) (p = 0.189).

### 3.4 Charge delivered at RMT intensity

Significant main effects of carrier frequency and waveform were observed for total charge delivered at average RMT (p < 0.001), along with significant interaction effects (p < 0.001).

**Fig. 5:**
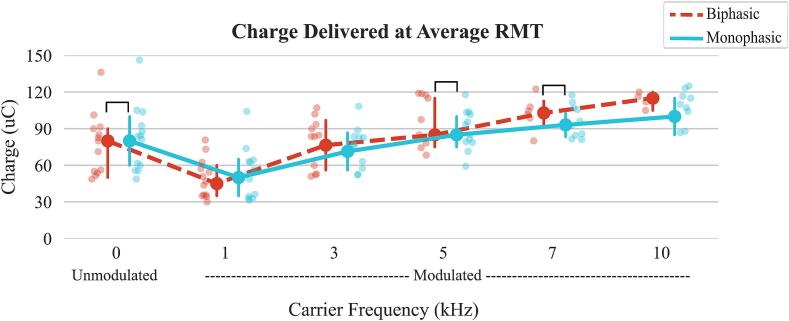
Charge delivered at RMT for each waveform-carrier frequency configuration. Charge was calculated by multiplying the current intensity delivered at RMT by the amount of time the current was delivered within that pulse. Brackets indicate a significant difference between monophasic and biphasic at a certain carrier frequency. The plot shows the median values, with error bars representing the interquartile range (IQR), covering the 25th to 75th percentiles of the data.

For total charge at the average RMT (Fig. 5), the charge of monophasic waveform was greater than biphasic at 0 kHz (M0: median 83.8 uC (IQR: 60.0–92.5) vs. B0: 80.0 uC (55.0–90.0), p = 0.017). However, at 5 kHz and 7 kHz carrier frequency, the charge was higher with biphasic waveform compared to monophasic waveform (B5: 97.5 uC (78.1–117.5) vs. M5: 89.1 uC (79.2–102.1), p < 0.001; B7: 103.0 uC (99.4–107.0) vs. M7: 94.3 uC (84.8–106.5), p = 0.001). See Supplementary Table S5 for all pairwise comparisons results.

### 3.5 Number of activated muscles

**Fig. 6:**
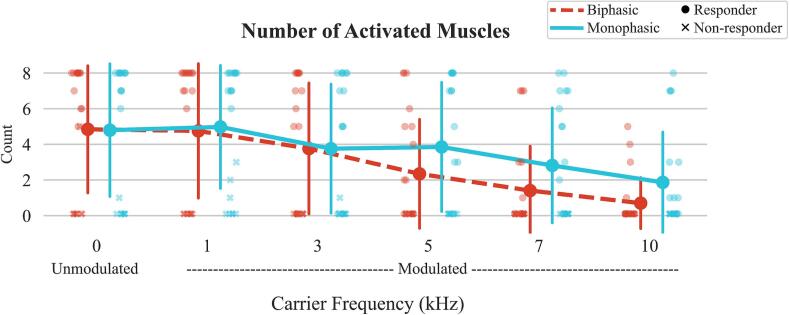
Number of muscles that reached an RMT (peak-to-peak amplitude > 0.05 mV) for each waveform-carrier frequency configuration. A maximum of 8 muscles could be activated. The plot shows the median values, with error bars representing the interquartile range (IQR), covering the 25th to 75th percentiles of the data. Eight of the twenty-one participants that were defined as “non-responders” since they did not have a muscle response (no RMT reached) for at least 4 out of 8 muscles in the 0 kHz configurations are marked with an “x”.

There was a significant interaction effect on the number of activated muscles (p = 0.001), however, no significance was found between relevant configurations, as shown in Fig. 6. In addition, there were also significant carrier frequency (p < 0.001) and waveform (p < 0.001) effects. In general, higher carrier frequencies activated less number of muscles. Additionally, biphasic activated less number of muscles compared to monophasic at higher carrier frequencies. Supplementary Table S6 contains all the p-values for the pairwise comparisons of number of muscles activated across all configurations.

Eight out of the 21 participants did not activate at least 4 muscles in all the waveforms. The single-pulse tolerance was significantly higher for responders than non-responders (39 % higher) for all waveform-frequency configurations (responders: median 250 mA (IQR: 160–250) vs non-responders: 140 mA (90–203), p < 0.001). Statistical analysis showed significant differences for single-pulse tolerance at B1 (responders: 150.0 mA (120.0–200.0) vs. 90.0 mA (75.0–100.0), p = 0.011), B3 (250.0 mA (190.0–250.0) vs. 125.0 mA (90.0–160.0), p = 0.004), M0 (140.0 mA (110.0–160.0) vs. 80.0 mA (65.0–92.5), p = 0.027), M7 (250.0 mA (210.0–250.0) vs. 185.0 mA (140.0–205.0), p = 0.047). A similar trend was also seen for continuous tolerance (responders: median 81 mA (IQR: 52–123), non-responders: 51 mA (28–80), p = 0.015). Additionally, 7 out of the 8 non-responders were female, which accounted for the 7 out of 9 women in our sample. Overall, women had a lower continuous tolerance compared to men (average tolerance women ± standard deviation: 65.4 ± 44.7 mA vs men: 97.5 ± 63.8 mA).

Lowest RMT, percent of lowest RMT tolerated, and charge delivered at lowest RMT results are presented in Supplementary Table S7-S9 and Supplementary Fig. S1.

## 4 Discussion

Selecting appropriate tSCS stimulation parameters is crucial for maximizing efficacy and establishing clinical standards in for application in post-stroke rehabilitation. Specifically, stimulation intensity used varies widely in the literature and may not be transferable across different waveform and frequency configurations. Additionally, comfort is a key consideration for feasibility especially in populations that may be more sensitive to stimulation.

This study evaluated how two commonly varied stimulation parameters, waveform and carrier frequency, influence motor thresholds, tolerance levels, charge delivery, and muscle activation in individuals post-stroke. We found that higher carrier frequencies increased RMTs, tolerance, and total charge delivered. Significant differences between monophasic and biphasic waveforms were observed only in certain configurations, with biphasic waveforms generally associated higher RMTs, tolerance, and charge delivered than monophasic waveforms— except for 0 kHz and 1 kHz. However, the percent of RMT tolerated remained consistent across configurations, suggesting that although absolute stimulation intensities vary, comfort relative to motor threshold remains stable. Notably, the results suggest that very high intensities, such as 90 % of average RMT, may be difficult to achieve across waveform-frequency configurations. We also found that both carrier frequency and waveform affected muscles activation, with higher carrier frequencies and biphasic waveforms associated with fewer muscles activated.

Together, these findings support ongoing development of tSCS protocols that are both effective and tolerable for individuals post-stroke. By clarifying how waveform and carrier frequency influence key physiological and perceptual outcomes, this study helps address existing gaps in the literature and provides a foundation for future study designs focused on utilizing tSCS for neurorehabilitation.

### 4.1 Resting motor threshold (RMT) changes with carrier frequency and waveform

#### 4.1.1 Effect of carrier frequency

Our results showed that both average and lowest RMTs increased with carrier frequency, along with the total charge delivered at RMT. This suggests that charge alone does not determine motor response—if it did, all configurations would require similar charge at RMT. For example, due to the 50 % duty cycle, 1–10 kHz waveforms would need roughly double the RMT intensity of unmodulated pulses to match charge, yet still required more to elicit responses. We hypothesize that rapid polarity shifts at higher frequencies disrupt charge integration at the membrane, reducing stimulation efficiency despite higher delivered charge. These findings align with prior studies: Manson et al. (2020) reported 1.4x more charge required at 5 kHz vs. unmodulated (Manson et al., 2020), similar to our 1.5x difference. Dalrymple et al. (2023) found a 1.9x increase at 10 kHz (Dalrymple et al., 2023); our data showed 1.7x for monophasic 10 kHz. Comparable trends have been observed in peripheral nerve stimulation (Luu et al., 2024). Overall, these results emphasize that stimulation parameters across configurations cannot be directly compared by charge alone, as equivalent charge does not produce equivalent motor responses.

#### 4.1.2 Effect of waveform

We also compared RMT differences between monophasic and biphasic waveforms. Our results indicated that monophasic waveforms tended to have higher average RMTs at unmodulated and 1 kHz carrier frequencies. The opposite was seen at higher carrier frequencies (5 kHz, 7 kHz, and 10 kHz), where more current was required to elicit a muscle response in biphasic compared to monophasic waveforms. This may be explained by the current reversal of a biphasic pulse depressing the developing graded potential that may cause eventually cause the depolarization level required for an action potential (Reilly, 2012).

### 4.2 Tolerance and discomfort changes with carrier frequency and waveform

#### 4.2.1 Effect of carrier frequency

We observed that both single-pulse and continuous tolerance increase with higher carrier frequencies for tSCS. Our results reveal that the introduction of carrier frequency plays a significant role in mitigating discomfort during tSCS at constant current intensities. This aligns with existing literature, which suggests that C-fibers are less likely to fire in response to high-frequency stimulation compared to larger-diameter fibers (Joseph and Butera, 2011, Ward and Chuen, 2009).

#### 4.2.2 Effect of waveform

In contrast, we did not find significant differences in tolerance between biphasic and monophasic waveforms when compared at constant carrier frequencies. While previous studies have recommended biphasic waveforms over monophasic waveforms due to discomfort considerations (Hofmann et al., 2011), and has been suggested they may activate different neural circuits (Wang et al., 2012), their impact on discomfort at the lumbar region appears to be comparable.

### 4.3 Percent of RMT tolerated, feasibility, and number of activated muscles

To assess comfort near motor threshold, we calculated the percentage of RMT participants could tolerate across all stimulation configurations. The purpose was to understand if there are more comfortable configurations that could be used to stimulate near the RMT. Results showed no significant differences in tolerated percentages across waveforms or within the same carrier frequency. Biphasic waveforms generally allowed higher tolerance, except at 10 kHz, and carrier frequency had minimal effect. On average, participants tolerated only 42–65 % of their RMT across configurations, indicating that continuous tSCS was generally subthreshold. Even for the lowest RMTs, tolerance ranged only from 48–85 %. This highlights the importance of reporting both RMT and stimulation intensity in future studies to guide safe and effective dosing. As more studies explore translating tSCS into different populations, the stimulation intensity used and the RMT should be recorded to have a reference of stimulation dosage during therapy. Such reports could help discern the optimal settings that balance comfort and therapeutic efficacy.

Muscle activation analysis revealed that unmodulated and 1 kHz stimulation activated the most muscles during sMERs, while higher carrier frequencies were less effective, especially with biphasic waveforms. Intensity during sMERs was capped at 250 mA, which could have limited activation but also emphasizes the importance of safety considerations in stimulation intensity protocols, especially since higher carrier frequencies required more charge to reach a response. These findings offer insights into design of stimulation equipment and protocols for new research laboratories aiming to study the use of tSCS. Our results suggest that introducing a carrier frequency for discomfort consideration may not be necessary. However, these configurations were evaluated based on motor thresholds and tolerance. More research on the therapeutic and rehabilitation effects of varying carrier frequency and/or waveform needs to be done.

Eight of the 21 participants were unable to reach measurable RMTs, primarily due to low stimulation tolerance—averaging 39 % lower than that of responders. These non-responders tolerated only 147 ± 67 mA, falling below the 157 mA average threshold needed to elicit a muscle response. Notably, most non-responders were women, suggesting greater sensitivity to stimulation discomfort, consistent with documented sex differences in pain sensitivity (Berkley, 1997). In addition to increased BMI (Veit et al., 2024), reduced tolerance may be a factor limiting the ability to determine RMTs in some individuals. Implementing a sMERs session at the start of a study could help assess participant responsiveness, eligibility, and promote acclimation, as we observed a 16 % increase in tolerance following habituation.

### 4.4 Study limitations

We acknowledge that the motor threshold, measured while participants were lying down, may change when transitioning to an upright posture or during walking (Danner et al., 2016, Megia-Garcia et al., 2021). As a result, calculating the actual dosage of current delivery based on motor threshold from standing may differ and more research needs to be done how this relates to the resting motor threshold and impacts “subthreshold” stimulation. Additionally, there are tSCS studies placing the electrode at different lumbar locations to target lower extremity motor function (Gerasimenko et al., 2015, Kumru et al., 2024), this variation might impact the relative thresholds of different muscles. We observed considerable between-subject variability of RMTs and tolerance in our results. To ensure consistency, the team documented the placement locations of the tSCS and EMG electrodes through photographs and measurements from body landmarks.

## 5 Conclusions

This study evaluated the effects of waveform and carrier frequency parameters on both RMT and stimulation tolerance, providing an essential basis for assessing the feasibility of these configurations in a neurological population, such as individuals post-stroke, who tipically retain sensation at the site of the stimulation. This understanding is crucial before optimizing each parameter for specific rehabilitation goals. We observed that the discomfort to stimulation remained consistent across all configurations when stimulating relative to the RMT. However, biphasic waveforms at higher carrier frequencies were less effective in activating muscles. This finding may be taken into consideration if the goal is to target as many muscles as possible in a stimulation intervention. Additionally, it points to potential differences in the mechanisms of action between configurations, warranting further research to understand their therapeutic effects. Caution is advised when using charge to standardize stimulation thresholds across different tSCS studies with varying parameters, as the rate of charge delivery varies depending on carrier frequency used. Delivering the same charge with different configurations does not necessarily produce the same magnitude of muscle response. Future studies should apply these insights to rehabilitation outcomes to understand neuromuscular recruitment differences and the therapeutic benefits associated with each waveform and carrier frequency configuration.

## Declaration of competing interest

The authors declare that they have no known competing financial interests or personal relationships that could have appeared to influence the work reported in this paper.
