# Supplementary material for: The effects of stimulation waveform and carrier frequency on tolerance and motor thresholds elicited by transcutaneous spinal cord stimulation in stroke
Source: Clin Neurophysiol Pract. 2025 Apr 22;10:150–8. doi: 10.1016/j.cnp.2025.04.001 (PMC12056781; doi:10.1016/j.cnp.2025.04.001)

**Supplemental material:**

**Supplementary Table 1. Average RMT (mA) ± standard deviation and number of muscles activated per participant and waveform-frequency configuration**

**Supplementary Table 2. Average RMT**

**Supplementary Table 3. Continuous stimulation tolerance per participant and waveform-frequency configuration (mA)**

**Supplementary Table 4. Percent of average RMT tolerated**

**Supplementary Table 5. Charge delivered at average RMT**

**Supplementary Table 6. Number of muscles activated**

**Supplementary Table 7. Lowest RMT**

**Supplementary Table 8. Percent of lowest RMT tolerated**

**Supplementary Table 9. Charge delivered at lowest RMT**

**Supplementary Figure 1. (A) Lowest RMT, (B) Percent of lowest RMT tolerated, and (C) Charge delivered at lowest RMT for all waveform-frequency configurations**

**Supplementary Table 1. Average RMT (mA) ± standard deviation and number of muscles activated per participant and waveform-frequency configuration:**

| **Participant ID** | **Waveform** | **0 kHz** | **1 kHz** | **3 kHz** | **5 kHz** | **7 kHz** | **10 kHz** |
| --- | --- | --- | --- | --- | --- | --- | --- |
| 1 | Biphasic | 90.0 ± 20.0  (n=8) | 146.2 ± 36.6 (n=8) | 166.0 ± 15.2 (n=5) | 230.0 ± 10.0 (n=5) |  |  |
|  | Monophasic | 92.5 ± 24.9  (n=8) | 104.3 ± 22.3 (n=7) | 162.0 ± 24.9 (n=5) | 203.3 ± 11.5 (n=3) | 240.0 ± 14.1 (n=2) | 246.7 ± 5.8  (n=3) |
| 2 | Biphasic | 80.0 ± 10.0  (n=5) | 108.8 ± 13.6 (n=8) | 164.0 ± 11.4 (n=5) | 235.0 ± 7.1  (n=2) |  |  |
|  | Monophasic | 84.3 ± 16.2  (n=7) | 126.2 ± 29.7 (n=8) | 162.5 ± 27.1 (n=8) | 191.2 ± 30.0 (n=8) | 228.0 ± 16.4 (n=5) | 250.0  (n=1) |
| 3 | Biphasic | 91.4 ± 3.8  (n=7) | 114.3 ± 7.9  (n=7) | 200.0 ± 45.4 (n=8) |  |  |  |
|  | Monophasic | 105.0 ± 10.7 (n=8) | 125.0 ± 13.1 (n=8) | 174.0 ± 8.9  (n=5) | 206.7 ± 15.3 (n=3) |  |  |
| 4 | Biphasic | 55.0 ± 12.2  (n=6) | 71.2 ± 15.5  (n=8) | 117.5 ± 16.7 (n=8) | 156.2 ± 16.9 (n=8) | 163.3 ± 5.8  (n=3) | 224.0 ± 19.5 (n=5) |
|  | Monophasic | 61.7 ± 9.8  (n=6) | 72.5 ± 13.9  (n=8) | 123.8 ± 25.6 (n=8) | 142.9 ± 26.3 (n=7) | 166.7 ± 20.8 (n=3) | 208.6 ± 34.4 (n=7) |
| 5 | Biphasic | 53.8 ± 13.0  (n=8) | 68.8 ± 21.7  (n=8) | 102.5 ± 11.6 (n=8) | 148.8 ± 23.6 (n=8) | 220.0 ± 16.3 (n=7) |  |
|  | Monophasic | 48.8 ± 6.4  (n=8) | 65.7 ± 5.3  (n=7) |  | 162.9 ± 12.5 (n=7) | 188.6 ± 10.7 (n=7) | 173.8 ± 19.2 (n=8) |
| 6 | Biphasic | 71.2 ± 9.9  (n=8) | 93.8 ± 11.9  (n=8) | 176.2 ± 25.0 (n=8) | 237.5 ± 15.0 (n=4) | 250.0  (n=1) |  |
|  | Monophasic | 83.8 ± 15.1  (n=8) | 128.8 ± 23.0 (n=8) | 163.8 ± 25.6 (n=8) | 186.2 ± 21.3 (n=8) | 215.7 ± 22.3 (n=7) | 220.0  (n=1) |
| 7 | Biphasic | 48.8 ± 6.4  (n=8) | 71.2 ± 9.9  (n=8) | 103.8 ± 11.9 (n=8) | 170.0 ± 30.7 (n=8) | 201.4 ± 25.4 (n=7) | 210.0 ± 26.5 (n=3) |
|  | Monophasic | 60.0 ± 10.7  (n=8) | 68.8 ± 12.5  (n=8) | 102.5 ± 13.9 (n=8) | 157.5 ± 20.5 (n=8) | 172.5 ± 24.9 (n=8) | 214.3 ± 19.9 (n=7) |
| 8 | Biphasic | 136.2 ± 22.0 (n=8) | 161.4 ± 24.1 (n=7) |  |  |  |  |
|  | Monophasic | 146.2 ± 25.0 (n=8) | 208.3 ± 31.9 (n=6) |  |  |  |  |
| 9 | Biphasic | 101.2 ± 17.3 (n=8) | 125.0 ± 15.1 (n=8) | 210.0 ± 16.3  (n=7) |  |  |  |
|  | Monophasic | 103.8 ± 15.1 (n=8) | 147.5 ± 16.7 (n=8) | 212.5 ± 16.7 (n=8) | 236.0 ± 8.9  (n=5) |  |  |
| 10 | Biphasic | 56.2 ± 19.2  (n=8) | 60.0 ± 10.7  (n=8) | 100.0 ± 11.5 (n=7) | 136.7 ± 15.1 (n=6) | 207.1 ± 22.1 (n=7) | 232.5 ± 9.6  (n=4) |
|  | Monophasic | 55.7 ± 5.3  (n=7) | 62.9 ± 4.9  (n=7) | 102.9 ± 11.1 (n=7) | 118.6 ± 13.5 (n=7) | 165.7 ± 15.1 (n=7) | 175.7 ± 15.1 (n=7) |
| 11 | Biphasic | 85.0 ± 14.1  (n=8) | 101.4 ± 15.7 (n=7) | 183.3 ± 28.0 (n=6) | 238.0 ± 13.0 (n=5) |  | 240.0  (n=1) |
|  | Monophasic | 87.5 ± 16.7  (n=8) | 112.5 ± 23.1 (n=8) | 150.0 ± 15.8 (n=5) | 207.5 ± 27.1 (n=8) | 218.0 ± 26.8 (n=5) | 230.0  (n=1) |
| 12 | Biphasic | 82.5 ± 13.9  (n=8) | 87.5 ± 27.1  (n=8) | 163.8 ± 29.7 (n=8) | 195.0 ± 7.1  (n=2) | 213.3 ± 11.5 (n=3) |  |
|  | Monophasic | 81.2 ± 11.3  (n=8) | 97.5 ± 15.8  (n=8) | 151.2 ± 18.1 (n=8) | 170.0 ± 26.2 (n=8) | 196.2 ± 23.3 (n=8) | 233.3 ± 15.3 (n=3) |
| 13 | Biphasic | 51.7 ± 7.5  (n=6) | 70.0 ± 12.6  (n=6) |  |  |  |  |
|  | Monophasic | 55.7 ± 7.9  (n=7) | 65.7 ± 5.3  (n=7) | 112.9 ± 4.9  (n=7) | 158.8 ± 8.3  (n=8) | 175.0 ± 17.6 (n=6) |  |

**Supplementary Table 2. Average RMT pairwise comparisons (n = 13):**

| **Average RMT - Monophasic with different carrier frequencies**  (Pairwise comparison Sequential Bonferroni p values) | | | | | | |
| --- | --- | --- | --- | --- | --- | --- |
|  | Unmodulated (0kHz) | 1 kHz | 3 kHz | 5 kHz | 7 kHz | 10 kHz |
| Unmodulated (0kHz) | **/** | **<0.001** | **<0.001** | **<0.001** | **<0.001** | **<0.001** |
| 1 kHz | **<0.001** | **/** | **<0.001** | **<0.001** | **<0.001** | **<0.001** |
| 3 kHz | **<0.001** | **<0.001** | **/** | **<0.001** | **<0.001** | **<0.001** |
| 5 kHz | **<0.001** | **<0.001** | **<0.001** | **/** | **<0.001** | **<0.001** |
| 7 kHz | **<0.001** | **<0.001** | **<0.001** | **<0.001** | **/** | **<0.001** |
| 10 kHz | **<0.001** | **<0.001** | **<0.001** | **<0.001** | **<0.001** | **/** |

| **Average RMT - Biphasic with different carrier frequencies**  (Pairwise comparison Sequential Bonferroni p values) | | | | | | |
| --- | --- | --- | --- | --- | --- | --- |
|  | Unmodulated (0kHz) | 1 kHz | 3 kHz | 5 kHz | 7 kHz | 10 kHz |
| Unmodulated (0kHz) | / | **<0.001** | **<0.001** | **<0.001** | **<0.001** | **<0.001** |
| 1 kHz | **<0.001** | / | **<0.001** | **<0.001** | **<0.001** | **<0.001** |
| 3 kHz | **<0.001** | **<0.001** | / | **<0.001** | **<0.001** | **<0.001** |
| 5 kHz | **<0.001** | **<0.001** | **<0.001** | / | **0.005** | **0.005** |
| 7 kHz | **<0.001** | **<0.001** | **<0.001** | **0.005** | / | 0.614 |
| 10 kHz | **<0.001** | **<0.001** | **<0.001** | **0.005** | 0.614 | / |

| **Average RMT – Same carrier frequency with different waveforms**  (Pairwise comparison Sequential Bonferroni p values) | | | | | | |
| --- | --- | --- | --- | --- | --- | --- |
|  | Unmodulated | 1 kHz | 3 kHz | 5 kHz | 7 kHz | 10 kHz |
| Biphasic-Monophasic | **0.012** | 0.614 | 0.461 | **<0.001** | **0.001** | 0.051 |

**Supplementary Table 3. Continuous stimulation tolerance per participant and waveform-frequency configuration (mA):**

| **Participant ID** | **Waveform** | **0 kHz** | **1 kHz** | **3 kHz** | **5 kHz** | **7 kHz** | **10 kHz** | |
| --- | --- | --- | --- | --- | --- | --- | --- | --- |
| 1 | Biphasic | 38 | 120 | 57 | 86 | 109 | 148 |  |
|  | Monophasic | 27 | 51 | 80 | 92 | 104 | 138 |  |
| 2 | Biphasic | 36 | 29 | 53 | 83 | 68 | 89 |  |
|  | Monophasic | 18 | 38 | 35 | 27 | 63 | 95 |  |
| 3 | Biphasic | 23 | 47 | 95 | 110 | 119 | 176 |  |
|  | Monophasic | 29 | 53 | 51 | 60 | 118 | 150 |  |
| 4 | Biphasic | 40 | 50 | 75 | 103 | 113 | 130 |  |
|  | Monophasic | 6 | 51 | 65 | 47 | 129 | 80 |  |
| 5 | Biphasic | 45 | 56 | 74 | 115 | 164 | 135 |  |
|  | Monophasic | 56 | 63 | 85 | 72 | 98 | 118 |  |
| 6 | Biphasic | 63 | 53 | 123 | 182 | 183 | 250 |  |
|  | Monophasic | 74 | 98 | 101 | 120 | 146 | 224 |  |
| 7 | Biphasic | 33 | 36 | 81 | 112 | 129 | 134 |  |
|  | Monophasic | 27 | 72 | 86 | 93 | 96 | 193 |  |
| 8 | Biphasic | 107 | 148 | 250 | 250 | 250 | 250 |  |
|  | Monophasic | 115 | 138 | 240 | 250 | 250 | 250 |  |
| 9 | Biphasic | 78 | 110 | 165 | 185 | 250 | 124 |  |
|  | Monophasic | 104 | 86 | 162 | 205 | 156 | 203 |  |
| 10 | Biphasic | 28 | 42 | 84 | 79 | 58 | 74 |  |
|  | Monophasic | 23 | 29 | 45 | 72 | 78 | 89 |  |
| 11 | Biphasic | 52 | 77 | 107 | 116 | 180 | 194 |  |
|  | Monophasic | 65 | 78 | 118 | 76 | 150 | 171 |  |
| 12 | Biphasic | 24 | 38 | 48 | 79 | 116 | 134 |  |
|  | Monophasic | 27 | 52 | 53 | 51 | 58 | 100 |  |
| 13 | Biphasic | 21 | 27 | 50 | 45 | 73 | 65 |  |
|  | Monophasic | 18 | 22 | 56 | 43 | 61 | 78 |  |
| 14 | Biphasic | 36 | 76 | 148 | 113 | 188 | 138 |  |
|  | Monophasic | 44 | 68 | 95 | 250 | 157 | 179 |  |
| 15 | Biphasic | 11 | 24 | 27 | 36 | 77 | 27 |  |
|  | Monophasic | 9 | 14 | 40 | 30 | 43 | 98 |  |
| 16 | Biphasic | 14 | 7 | 15 | 67 | 138 | 156 |  |
|  | Monophasic | 2 | 28 | 28 | 100 | 103 | 90 |  |
| 17 | Biphasic | 14 | 23 | 35 | 26 | 62 | 32 |  |
|  | Monophasic | 12 | 22 | 26 | 22 | 71 | 40 |  |
| 18 | Biphasic | 12 | 45 | 40 | 74.2 | 79 | 58 |  |
|  | Monophasic | 29 | 38 | 58 | 58 | 65 | 62 |  |
| 19 | Biphasic | 23 | 48 | 77 | 62 | 113 | 122 |  |
|  | Monophasic | 33 | 54 | 70 | 81 | 62 | 68 |  |
| 20 | Biphasic | 50 | 24 | 50 | 44 | 100 | 84 |  |
|  | Monophasic | 18 | 20 | 38 | 57 | 113 | 143 |  |
| 21 | Biphasic | 15 | 48 | 80 | 46 | 89 | 86 |  |
|  | Monophasic | 31 | 60 | 50 | 51 | 70 | 115 |  |

**Supplementary Table 4. Percent of average RMT tolerated (n = 13):**

| **Percent of average RMT tolerated - Monophasic with different carrier frequencies**  (Pairwise comparison Sequential Bonferroni p values) | | | | | | |
| --- | --- | --- | --- | --- | --- | --- |
|  | Unmodulated (0kHz) | 1 kHz | 3 kHz | 5 kHz | 7 kHz | 10 kHz |
| Unmodulated (0kHz) | / | >0.99 | >0.99 | >0.99 | >0.99 | >0.99 |
| 1 kHz | >0.99 | / | >0.99 | >0.99 | >0.99 | >0.99 |
| 3 kHz | >0.99 | >0.99 | / | >0.99 | >0.99 | >0.99 |
| 5 kHz | >0.99 | >0.99 | >0.99 | / | >0.99 | 0.278 |
| 7 kHz | >0.99 | >0.99 | >0.99 | >0.99 | / | >0.99 |
| 10 kHz | >0.99 | >0.99 | >0.99 | 0.278 | >0.99 | / |

| **Percent of average RMT tolerated - Biphasic with different carrier frequencies**  (Pairwise comparison Sequential Bonferroni p values) | | | | | | |
| --- | --- | --- | --- | --- | --- | --- |
|  | Unmodulated (0kHz) | 1 kHz | 3 kHz | 5 kHz | 7 kHz | 10 kHz |
| Unmodulated (0kHz) | / | >0.99 | >0.99 | >0.99 | >0.99 | >0.99 |
| 1 kHz | >0.99 | / | >0.99 | >0.99 | >0.99 | >0.99 |
| 3 kHz | >0.99 | >0.99 | / | >0.99 | >0.99 | >0.99 |
| 5 kHz | >0.99 | >0.99 | >0.99 | / | >0.99 | >0.99 |
| 7 kHz | >0.99 | >0.99 | >0.99 | >0.99 | / | >0.99 |
| 10 kHz | >0.99 | >0.99 | >0.99 | >0.99 | >0.99 | / |

| **Percent of average RMT tolerated – Same carrier frequency with different waveforms**  (Pairwise comparison Sequential Bonferroni p values) | | | | | | |
| --- | --- | --- | --- | --- | --- | --- |
|  | Unmodulated | 1 kHz | 3 kHz | 5 kHz | 7 kHz | 10 kHz |
| Biphasic-Monophasic | >0.99 | >0.99 | >0.99 | 0.723 | >0.99 | >0.99 |

**Supplementary Table 5. Charge delivered at average RMT (n = 13):**

| **Charge delivered at average RMT - Monophasic with different carrier frequencies**  (Pairwise comparison Sequential Bonferroni p values) | | | | | | |
| --- | --- | --- | --- | --- | --- | --- |
|  | Unmodulated (0kHz) | 1 kHz | 3 kHz | 5 kHz | 7 kHz | 10 kHz |
| Unmodulated (0kHz) | / | **<0.001** | 0.527 | **<0.001** | **<0.001** | **<0.001** |
| 1 kHz | **<0.001** | / | **<0.001** | **<0.001** | **<0.001** | **<0.001** |
| 3 kHz | 0.527 | **<0.001** | / | **<0.001** | **<0.001** | **<0.001** |
| 5 kHz | **<0.001** | **<0.001** | **<0.001** | / | **<0.001** | **<0.001** |
| 7 kHz | **<0.001** | **<0.001** | **<0.001** | **<0.001** | / | **0.004** |
| 10 kHz | **<0.001** | **<0.001** | **<0.001** | **<0.001** | **0.004** | / |

| **Charge delivered at average RMT - Biphasic with different carrier frequencies**  (Pairwise comparison Sequential Bonferroni p values) | | | | | | |
| --- | --- | --- | --- | --- | --- | --- |
|  | Unmodulated (0kHz) | 1 kHz | 3 kHz | 5 kHz | 7 kHz | 10 kHz |
| Unmodulated (0kHz) | / | **<0.001** | 0.280 | **<0.001** | **<0.001** | **<0.001** |
| 1 kHz | **<0.001** | / | **<0.001** | **<0.001** | **<0.001** | **<0.001** |
| 3 kHz | 0.280 | **<0.001** | / | **<0.001** | **<0.001** | **<0.001** |
| 5 kHz | **<0.001** | **<0.001** | **<0.001** | / | **0.022** | **0.007** |
| 7 kHz | **<0.001** | **<0.001** | **<0.001** | **0.022** | / | 0.645 |
| 10 kHz | **<0.001** | **<0.001** | **<0.001** | **0.007** | 0.645 | / |

| **Charge delivered at average RMT – Same carrier frequency with different waveforms**  (Pairwise comparison Sequential Bonferroni p values) | | | | | | |
| --- | --- | --- | --- | --- | --- | --- |
|  | Unmodulated | 1 kHz | 3 kHz | 5 kHz | 7 kHz | 10 kHz |
| Biphasic-Monophasic | **0.017** | 0.767 | 0.645 | **<0.001** | **0.001** | 0.087 |

**Supplementary Table 6. Number of activated muscles (n = 21):**

| **Number of activated muscles - Monophasic with different carrier frequencies**  (Pairwise comparison Sequential Bonferroni p values) | | | | | | |
| --- | --- | --- | --- | --- | --- | --- |
|  | Unmodulated (0kHz) | 1 kHz | 3 kHz | 5 kHz | 7 kHz | 10 kHz |
| Unmodulated (0kHz) | / | >0.99 | >0.99 | >0.99 | 0.051 | **0.004** |
| 1 kHz | >0.99 | / | 0.183 | 0.185 | **0.008** | **<0.001** |
| 3 kHz | >0.99 | 0.183 | / | >0.99 | >0.99 | 0.424 |
| 5 kHz | >0.99 | 0.185 | >0.99 | / | 0.076 | 0.083 |
| 7 kHz | 0.051 | **0.008** | >0.99 | 0.076 | / | >0.99 |
| 10 kHz | **0.004** | **<0.001** | 0.424 | 0.083 | >0.99 | / |

| **Number of activated muscles - Biphasic with different carrier frequencies**  (Pairwise comparison Sequential Bonferroni p values) | | | | | | |
| --- | --- | --- | --- | --- | --- | --- |
|  | Unmodulated (0kHz) | 1 kHz | 3 kHz | 5 kHz | 7 kHz | 10 kHz |
| Unmodulated (0kHz) | / | >0.99 | 0.890 | **0.006** | **<0.001** | **<0.001** |
| 1 kHz | >0.99 | / |  | **0.007** | **<0.001** | **<0.001** |
| 3 kHz | 0.890 | 0.600 | / | 0.275 | **0.009** | **0.001** |
| 5 kHz | **0.006** | **0.007** | 0.275 | / | 0.527 | **0.036** |
| 7 kHz | **<0.001** | **<0.001** | **0.009** | 0.527 | / | >0.99 |
| 10 kHz | **<0.001** | **<0.001** | **0.001** | **0.036** | >0.99 | / |

| **Number of activated muscles – Same carrier frequency with different waveforms**  (Pairwise comparison Sequential Bonferroni p values) | | | | | | |
| --- | --- | --- | --- | --- | --- | --- |
|  | Unmodulated | 1 kHz | 3 kHz | 5 kHz | 7 kHz | 10 kHz |
| Biphasic-Monophasic | >0.99 | >0.99 | >0.99 | 0.287 | 0.146 | 0.204 |

**Supplementary Table 7. Lowest RMT (n = 13):**

| **Lowest RMT - Monophasic with different carrier frequencies**  (Pairwise comparison Sequential Bonferroni p values) | | | | | | |
| --- | --- | --- | --- | --- | --- | --- |
|  | Unmodulated (0kHz) | 1 kHz | 3 kHz | 5 kHz | 7 kHz | 10 kHz |
| Unmodulated (0kHz) | / | **<0.001** | **<0.001** | **<0.001** | **<0.001** | **<0.001** |
| 1 kHz | **<0.001** | / | **<0.001** | **<0.001** | **<0.001** | **<0.001** |
| 3 kHz | **<0.001** | **<0.001** | / | **<0.001** | **<0.001** | **<0.001** |
| 5 kHz | **<0.001** | **<0.001** | **<0.001** | / | **<0.001** | **<0.001** |
| 7 kHz | **<0.001** | **<0.001** | **<0.001** | **<0.001** | / | **0.002** |
| 10 kHz | **<0.001** | **<0.001** | **<0.001** | **<0.001** | **0.002** | / |

| **Lowest RMT - Biphasic with different carrier frequencies**  (Pairwise comparison Sequential Bonferroni p values) | | | | | | |
| --- | --- | --- | --- | --- | --- | --- |
|  | Unmodulated (0kHz) | 1 kHz | 3 kHz | 5 kHz | 7 kHz | 10 kHz |
| Unmodulated (0kHz) | / | **0.005** | **<0.001** | **<0.001** | **<0.001** | **<0.001** |
| 1 kHz | **0.005** | / | **<0.001** | **<0.001** | **<0.001** | **<0.001** |
| 3 kHz | **<0.001** | **<0.001** | / | **<0.001** | **<0.001** | **<0.001** |
| 5 kHz | **<0.001** | **<0.001** | **<0.001** | / | **<0.001** | **<0.001** |
| 7 kHz | **<0.001** | **<0.001** | **<0.001** | **<0.001** | / | 0.141 |
| 10 kHz | **<0.001** | **<0.001** | **<0.001** | **<0.001** | 0.141 | / |

| **Lowest RMT – Same carrier frequency with different waveforms**  (Pairwise comparison Sequential Bonferroni p values) | | | | | | |
| --- | --- | --- | --- | --- | --- | --- |
|  | Unmodulated | 1 kHz | 3 kHz | 5 kHz | 7 kHz | 10 kHz |
| Biphasic-Monophasic | 0.128 | 0.068 | >0.99 | **0.002** | **<0.001** | **0.011** |

**Supplementary Table 8. Percent of lowest RMT tolerated (n = 13):**

| **Percent of lowest RMT tolerated - Monophasic with different carrier frequencies**  (Pairwise comparison Sequential Bonferroni p values) | | | | | | |
| --- | --- | --- | --- | --- | --- | --- |
|  | Unmodulated (0kHz) | 1 kHz | 3 kHz | 5 kHz | 7 kHz | 10 kHz |
| Unmodulated (0kHz) | / | >0.99 | >0.99 | >0.99 | >0.99 | >0.99 |
| 1 kHz | >0.99 | / | >0.99 | 0.138 | 0.492 | >0.99 |
| 3 kHz | >0.99 | >0.99 | / | >0.99 | >0.99 | >0.99 |
| 5 kHz | >0.99 | 0.138 | >0.99 | / | >0.99 | >0.99 |
| 7 kHz | >0.99 | 0.492 | >0.99 | >0.99 | / | >0.99 |
| 10 kHz | >0.99 | >0.99 | >0.99 | >0.99 | >0.99 | / |

| **Percent of lowest RMT tolerated - Biphasic with different carrier frequencies**  (Pairwise comparison Sequential Bonferroni p values) | | | | | | |
| --- | --- | --- | --- | --- | --- | --- |
|  | Unmodulated (0kHz) | 1 kHz | 3 kHz | 5 kHz | 7 kHz | 10 kHz |
| Unmodulated (0kHz) | / | >0.99 | >0.99 | 0.179 | >0.99 | >0.99 |
| 1 kHz | >0.99 | / | >0.99 | >0.99 | 0.152 | 0.247 |
| 3 kHz | >0.99 | >0.99 | / | >0.99 | >0.99 | >0.99 |
| 5 kHz | 0.179 | >0.99 | >0.99 | / | >0.99 | >0.99 |
| 7 kHz | >0.99 | 0.152 | >0.99 | >0.99 | / | >0.99 |
| 10 kHz | >0.99 | 0.247 | >0.99 | >0.99 | >0.99 | / |

| **Percent of lowest RMT tolerated – Same carrier frequency with different waveforms**  (Pairwise comparison Sequential Bonferroni p values) | | | | | | |
| --- | --- | --- | --- | --- | --- | --- |
|  | Unmodulated | 1 kHz | 3 kHz | 5 kHz | 7 kHz | 10 kHz |
| Biphasic-Monophasic | >0.99 | >0.99 | >0.99 | >0.99 | >0.99 | >0.99 |

**Supplementary Table 9. Charge delivered at lowest RMT (n = 13):**

| **Charge delivered at lowest RMT - Monophasic with different carrier frequencies**  (Pairwise comparison Sequential Bonferroni p values) | | | | | | |
| --- | --- | --- | --- | --- | --- | --- |
|  | Unmodulated (0kHz) | 1 kHz | 3 kHz | 5 kHz | 7 kHz | 10 kHz |
| Unmodulated (0kHz) | / | **<0.001** | >0.99 | **<0.001** | **<0.001** | **<0.001** |
| 1 kHz | **<0.001** | / | **<0.001** | **<0.001** | **<0.001** | **<0.001** |
| 3 kHz | >0.99 | **<0.001** | / | **<0.001** | **<0.001** | **<0.001** |
| 5 kHz | **<0.001** | **<0.001** | **<0.001** | / | **<0.001** | **<0.001** |
| 7 kHz | **<0.001** | **<0.001** | **<0.001** | **<0.001** | / | **<0.001** |
| 10 kHz | **<0.001** | **<0.001** | **<0.001** | **<0.001** | **<0.001** | / |

| **Charge delivered at lowest RMT - Biphasic with different carrier frequencies**  (Pairwise comparison Sequential Bonferroni p values) | | | | | | |
| --- | --- | --- | --- | --- | --- | --- |
|  | Unmodulated (0kHz) | 1 kHz | 3 kHz | 5 kHz | 7 kHz | 10 kHz |
| Unmodulated (0kHz) | / | **<0.001** | >0.99 | **<0.001** | **<0.001** | **<0.001** |
| 1 kHz | **<0.001** | / | **<0.001** | **<0.001** | **<0.001** | **<0.001** |
| 3 kHz | >0.99 | **<0.001** | / | **<0.001** | **<0.001** | **<0.001** |
| 5 kHz | **<0.001** | **<0.001** | **<0.001** | / | **<0.001** | **<0.001** |
| 7 kHz | **<0.001** | **<0.001** | **<0.001** | **<0.001** | / | 0.128 |
| 10 kHz | **<0.001** | **<0.001** | **<0.001** | **<0.001** | 0.128 | / |

| **Charge delivered at lowest RMT – Same carrier frequency with different waveforms**  (Pairwise comparison Sequential Bonferroni p values) | | | | | | |
| --- | --- | --- | --- | --- | --- | --- |
|  | Unmodulated | 1 kHz | 3 kHz | 5 kHz | 7 kHz | 10 kHz |
| Biphasic-Monophasic | 0.205 | 0.098 | >0.99 | **0.002** | **<0.001** | 0.051 |

**Supplementary Figure 1.** (A) Lowest RMT, (B) percentage tolerated, and (C) charge delivered at the lowest RMT. Corresponding pairwise comparisons can be found in Supplementary tables 7, 8, and 9 respectively. The plot shows the median values, with error bars representing the interquartile range (IQR), covering the 25th to 75th percentiles of the data. RMT: resting motor threshold.


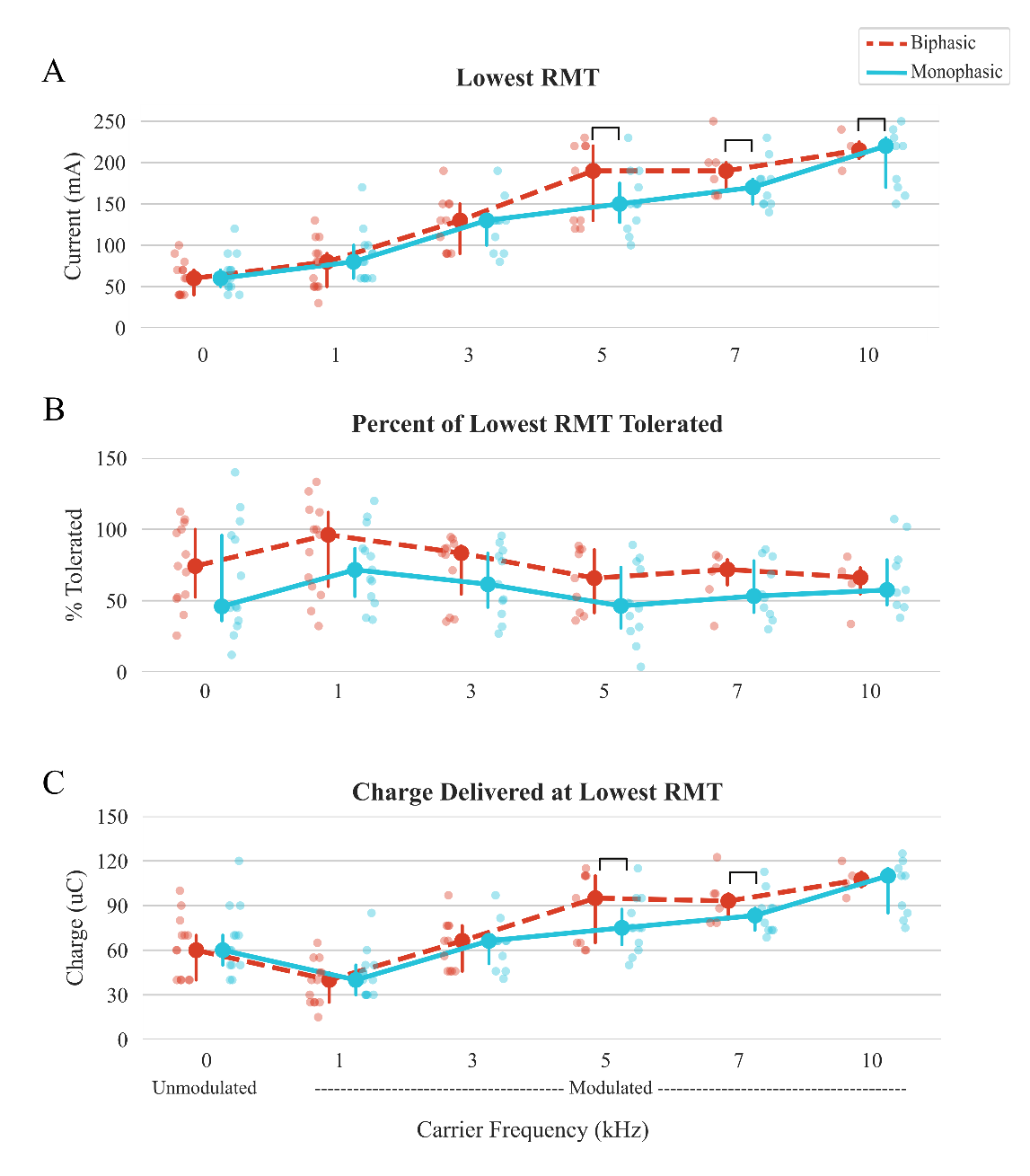

Supplement: Supplementary Data 1 [file mmc1.docx]
